# Mesenchymal tumours of the mediastinum—part II

**DOI:** 10.1007/s00428-015-1832-6

**Published:** 2015-09-10

**Authors:** Michael A. den Bakker, Alexander Marx, Kiyoshi Mukai, Philipp Ströbel

**Affiliations:** Department of Pathology, Maasstad Ziekenhuis, PO Box 9100, 3007 AC Rotterdam, The Netherlands; Department of Pathology, Erasmus MC, Rotterdam, The Netherlands; Institute of Pathology, University Medical Center Mannheim, University of Heidelberg, Heidelberg, Germany; Department of Diagnostic Pathology, Saiseikai Central Hospital, Tokyo, Japan; Department of Pathology, Universitätsmedizin Göttingen, Göttingen, Germany

**Keywords:** Mediastinum, Mesenchymal tumours, Soft tissue tumours

## Abstract

This is the second part of a two-part review on soft tissue tumours which may be encountered in the mediastinum. This review is based on the 2013 WHO classification of soft tissue tumours and the 2015 WHO classification of tumours of the lung, pleura, thymus and heart and provides an updated overview of mesenchymal tumours that have been reported in the mediastinum.

## Introduction

In this second part of a two-part review, the 2013 WHO classification of soft tissue tumours and the 2015 WHO classification of tumours of the thymus form the basis to summarize current knowledge about soft tissue tumours in the mediastinum. This comprehensive review covers published reports from the English and Japanese literature. In the first of these reviews, general aspects, adipocytic, fibroblastic/myofibroblastic, fibrohistiocytic tumours and soft tissue tumours arising as somatic components in germ cell tumours, were discussed. We now turn to mediastinal smooth muscle, skeletal muscle, vascular, chondro-osseous, nerve sheath and miscellaneous tumours of uncertain differentiation, including undifferentiated sarcomas. Since most of the tumours described here closely resemble soft tissue tumours elsewhere in the body, we will focus our review on defining criteria, epidemiology, clinical findings and prognosis, rather than histological descriptions. For in-depth coverage of the histological aspects of these tumours, the reader is referred to specific literature.

### Smooth muscle tumours

Smooth muscle cell tumours resemble normal smooth muscle and retain many of their immunohistochemical markers. Fewer benign smooth muscle cell tumours (leiomyomas) than malignant smooth muscle tumours (leiomyosarcomas) have been reported in the mediastinum.

To qualify as a primary mediastinal leiomyoma, tumours should arise from the somatic soft tissue of the mediastinum, whilst esophageal and large vessel primaries should be excluded. If these criteria are applied, primary mediastinal leiomyoma is very rare with approximately 20 reported cases in the literature [14, 19, 136, 139, 149, 162, 180, 205, 227]. Mediastinal leiomyoma is twice as common in females as in males and is mainly seen in the posterior mediastinum of older adults (age range 23–75 years; median age 50 years). These tumours may remain asymptomatic, or patients may complain of dyspnea or chest pain. The tumours may grow to a large size (up to 1,600 g). The morphology of mediastinal leiomyoma is similar to its counterparts elsewhere in the body and is defined as a neoplasm resembling normal smooth muscle cells and devoid of pleomorphism and necrosis and with a very low mitotic rate (<1 mitoses/50 HPF). Immunohistochemical stains will show positivity for smooth muscle actin and desmin in the majority of cases. Surgical removal is curative, and in the reports with follow-up, no recurrences or deaths due to tumour were described.

The main differential diagnosis of leiomyoma is leiomyosarcoma, which is distinguished by increased mitotic activity, pleomorphism and necrosis.

Mediastinal leiomyosarcoma is a very rare tumour with less than 40 cases described as case reports and in two small series [2, 13, 34, 37, 52, 72, 79, 82, 90, 106, 107, 111, 133, 163, 187, 188, 207, 228]. Mediastinal leiomyosarcoma occurs in adult patients (26–88 years) of either sex. A disproportionate number of cases arise in the posterior mediastinum, where tumours are often clinically asymptomatic. Resected tumours measured up to 18 cm, often without clear relationship to neighbouring anatomic structures. In some cases, an origin from major vessels seemed likely [188, 228], whilst in others, major vessels appeared rather entrapped within the tumour. Mediastinal leiomyosarcoma is typically a non-encapsulated, circumscribed mass, which may infiltrate the heart, lungs, thoracic vertebrae or spinal canal [34, 37, 107, 133]. Patients were treated by surgical resection, sometimes combined with chemo- and/or radiotherapy. Local recurrence and distant metastasis occur in a significant proportion of patients. The differential diagnosis of leiomyosarcoma comprises other spindle cell sarcomas, in particular synovial sarcoma and malignant peripheral nerve sheath tumours (MPNST). Whilst well-differentiated leiomyosarcoma will generally stain for both smooth muscle actin and desmin, less differentiated cases may show diminished staining or even absence of muscular markers, requiring additional investigations (e.g. calponin, caldesmon, TLE, S-100 protein and/or nestin immunohistochemistry or fluorescence in situ hybridisation (FISH) analyses) to rule out MPNST or synovial sarcoma. Rare cases of liposarcoma may contain a smooth muscle component (‘lipoleiomyosarcoma’) [62, 68]. In these cases, the lipomatous and smooth muscle components are admixed, and the dual differentiation is obvious. Leiomyosarcoma as a secondary non-germ cell sarcoma is extremely rare, and leiomyosarcoma has not been described as the sole mesenchymal component [35].

## Pericytic (perivascular) tumours

Less than ten cases of glomus tumour, including a single malignant variant, have been described in the mediastinum [20, 22, 30, 55, 64, 83, 108]. All cases were located in the posterior or superior compartments of the mediastinum in young or middle-aged females. The single malignant case, which resulted in this patient’s death, occurred in a 74-year-old female. Pain and dyspnea or cough were presenting symptoms. All benign cases were successfully treated by surgery without evidence of disease at follow-up.

A single case of (infantile) myofibromatosis, which in the WHO classification is included in the spectrum of pericytic tumours, located in the central mediastinum and involving the hilar region of the lung was described in a 4-year-old male infant and was treated by pneumonectomy [181].

Angioleiomyoma, which is included in the pericytic group of tumours in the current WHO classification, is considered a benign proliferation of vascular smooth muscle cells and has on rare occasion been reported in the mediastinum [85, 123, 161, 209, 225]. The reported tumours were (with one exception) small and were either discovered incidentally or caused symptoms by compression of nerves in the posterior mediastinum. In reports with follow-up, angioleiomyoma behaved in a benign fashion.

## Skeletal muscle tumours

Mediastinal mesenchymal tumours with skeletal muscle differentiation include rhabdomyoma, rhabdomyosarcoma, rhabdomyosarcoma as a component of MPNST (so-called Triton tumour) or as a somatic-type malignancy in germ cell tumours.

Rhabdomyoma is an uncommon benign skeletal muscle tumour. In the paediatric population, it is considered a hamartoma arising in the heart of children with tuberous sclerosis. Five cases of extra-cardiac non-syndromic rhabdomyoma have been described in the mediastinum. All patients were elderly adults (range 52–80 years; four males, one female) [21, 101, 126, 182, 233]. Symptomatic cases presented with non-specific signs. All cases had at some connection with the cervical area, one case was multifocal with a second cervical tumour [233]. It has been suggested that rhabdomyoma may originate from the third and fourth branchial pouches or from myoid cells in the thymus [182].

Primary mediastinal rhabdomyosarcoma is exceedingly rare. The nine reported cases with adequate documentation were all located in the anterior mediastinum [17, 31, 152, 160, 175, 192]. Seven cases occurred in males. Five cases were observed in young adults, two cases in children (4 months and 9 years of age) [31, 175] and two in older individuals. Six cases were diagnosed as alveolar rhabdomyosarcoma, which was confirmed by genetic analysis in two cases [31, 175]. Two cases were of the embryonal subtype, and one case was diagnosed as pleomorphic rhabdomyosarcoma. In the older literature, additional sparse reports of mediastinal rhabdomyosarcoma are on record. However, these cases predate immunohistochemical and molecular typing and cannot be reliably accepted as rhabdomyosarcomas [192]. The prognosis of the reported cases of primary mediastinal rhabdomyosarcoma was poor.

The differential diagnosis of the rare primary mediastinal rhabdomyosarcoma is the much more common rhabdomyosarcoma as a component of a germ cell tumour [23, 35, 69, 120]. In germ cell tumours with somatic-type malignancy, the prognosis is determined by the somatic (i.e. sarcomatous) component and in the setting of rhabdomyosarcoma is dismal. Rhabdomyoblastic differentiation has been reported in both low-grade (thymoma) [29, 129, 174] and high-grade (thymic carcinoma, carcinosarcoma) [41, 48, 145] thymic epithelial tumours and thus enters the differential diagnosis of rhabdomyosarcoma [29, 41, 48, 129, 145, 174]. Identification of an epithelial component by appropriate immunohistochemical staining will aid the accurate diagnosis. Rhabdomyoblastic differentiation may also occur in MPNST, which are then called malignant Triton tumours. Nine cases have been reported in the mediastinum (reviewed by Ren et al. [166]). The typical spindle cell MPSNT component usually predominates, whilst cells with rhabdomyoblastic differentiation commonly form a minor component of the tumour. These tumours carry a very poor prognosis.

## Vascular tumours

Amongst mesenchymal neoplasms in the mediastinum, vascular (endothelial) tumours are not uncommon with well over 100 cases reported in the literature.

### Benign and intermediate-grade vascular tumours

Benign blood vessel tumours (hemangioma variants) have been reported as individual cases and have been summarized in several reviews [32, 39, 127, 130]. Endothelial tumours of intermediate grade (hemangioendotheliomas) are characterized by local infiltrative growth and rare metastases.

Mediastinal hemangioma may originate in the soft tissue of the mediastinum or may arise in the thymus [5, 32, 154]. Mediastinal hemangiomas span a wide age range, from the newborn to the elderly, with most cases occurring in young adults. Of the reported cases, 50–75 % were located in the anterior mediastinum [32, 130]. Up to 50 % of cases were asymptomatic. Patients with symptomatic tumours presented with pain, cough and rarely with signs of compression of vital structures by the tumour, such as Horner’s syndrome, neurological signs and superior vena cava obstruction [32]. Tumour size ranged from a few centimetres to 20 cm [32, 130] and included both capillary and cavernous hemangioma subtypes. In the series by Moran and Suster, associated histological features included fatty metaplasia, fibrosis, smooth muscle overgrowth and inflammation [130]. Surgical resection is curative in most cases, although recurrence may rarely occur [32].

**Fig. 1:**
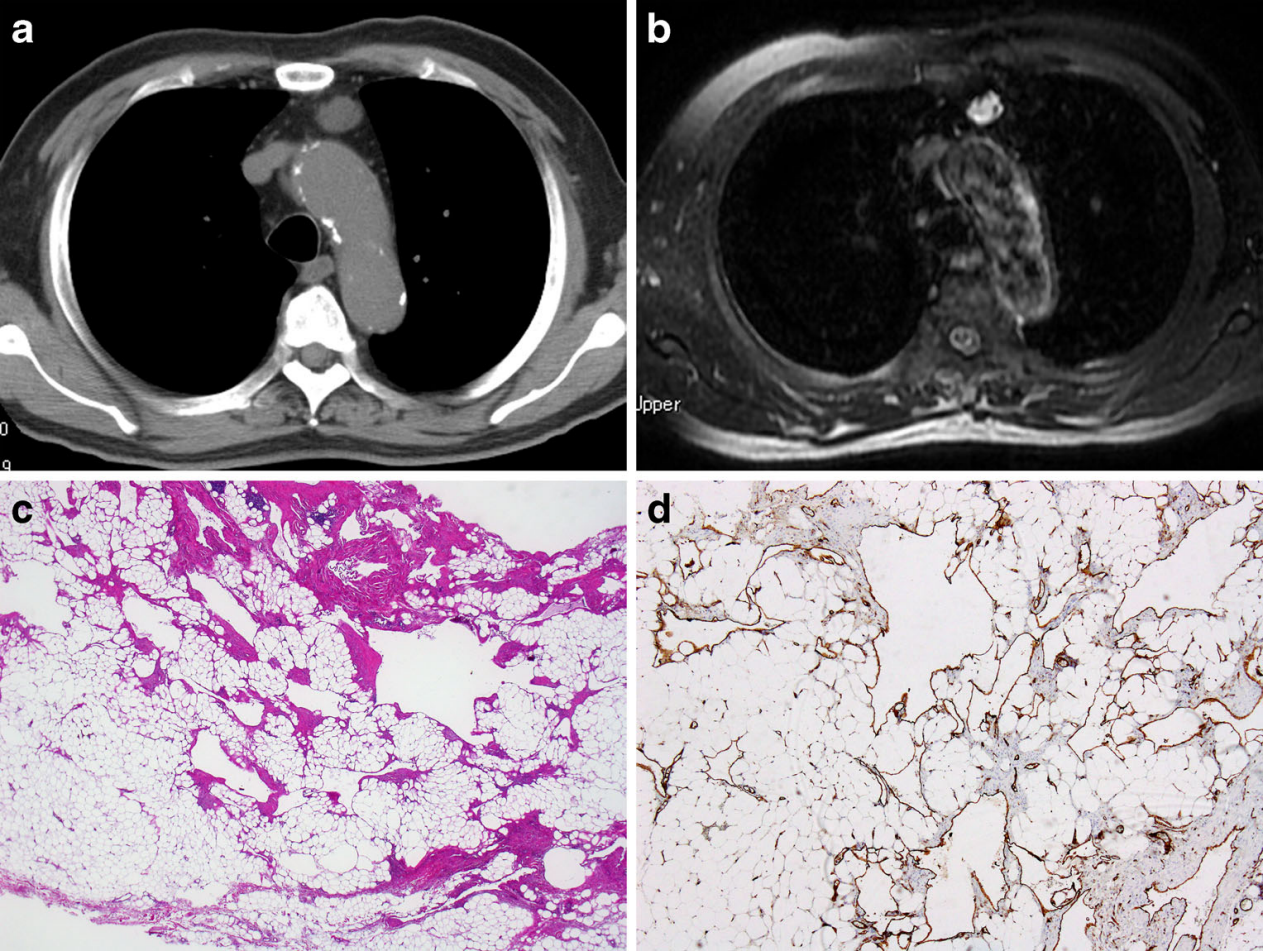
Lymphangioma. Anterior mediastinal mass considered to be a benign cystic lesion was discovered during routine check-up in an 81-year-old male. The mass increased in size from 16 to 22 mm in a period of 8 years. No recurrence 1 year after the surgery. **a** Non-enhanced CT axial image shows a small homogeneous mass with attenuation. **b** Axial fat-saturated T2-weighted MR image shows a hyperintense signal with small internal septations. **c** HE stain of resected tumour. Irregular thin-walled vascular structures in adipose tissue. **d** D2-40 stain, supporting the lymphatic differentiation of the lining endothelium

Lymphangioma has been reported as a cystic mass in the mediastinum of children and is very uncommon in adults (Fig. 1)

[61, 147, 156]. Lymphangiomas are not considered true neoplasms but rather malformations of the lymphatic vasculature with progressive cystic change resulting from obstruction of lymphatic drainage [80]. The progressive growth of these lesions causes dyspnea. Surgical resection is curative in most cases, although recurrences may require repeated surgery [156].

Vascular endothelial tumours of intermediate malignancy (with local aggressive behaviour and/or rare metastases) have been reported in the mediastinum as case reports and case series. These include kaposiform hemangioendothelioma (KHE) and a single case of composite hemangioendothelioma [24].

Less than 15 cases of exclusively mediastinal KHE have been reported, in addition to a number of cases where large KHEs extended contiguously from the neck into the mediastinum [51, 77, 81, 89, 114, 140, 210, 211, 220, 226, 234]. The reported mediastinal KHE cases were exclusively seen in infants (0–16 months of age) with almost equal sex distribution. Thrombocytopenic consumptive coagulopathy (Kasabach-Merritt syndrome) was present in almost all of the reported cases and is a major cause of fatality in this tumour. Mediastinal KHE is histologically similar to its soft tissue counterparts and is characterized by poorly circumscribed nodules of tightly packed small capillary-sized vessels, Kaposi sarcoma-like areas with spindled cells and absence of HHV-8 immunoreactivity. A component of larger lymphatic vessels is often present. The prognosis of KHE is variable. Complete surgical removal may provide a cure but may not be possible in cases with extensive infiltration. Variable success has been achieved by medical treatment with interferon and chemotherapy. The prognosis is often determined by the haematological complications rather than the local tumour effects. In the mediastinal cases with follow-up information, six patients were alive without evidence of disease, two patients were alive with disease and two patients died of disease.

A single case of composite hemangioendothelioma, a rare vascular neoplasm of intermediate grade with variable histological components, was described in the central mediastinal area of a 50-year-old female. There was evidence of disease 13 months after surgical resection [24]. 

### Malignant vascular tumours

Malignant vascular tumours comprising epithelioid hemangioendothelioma (EHE) and angiosarcoma (AS) have been reported in the mediastinum.

Mediastinal EHE is a malignant endothelial neoplasm with approximately 30 cases published mostly as case reports [7, 16, 25, 27, 56, 60, 88, 103, 109, 115, 121, 143, 151, 191, 202, 215, 216, 229] and a single series of 12 primary mediastinal cases described by Suster et al. [191] All cases occurred in adults (median age 46 years, age range 19–69 years) with twice as many cases reported in males as in females. A considerable proportion of EHE was discovered incidentally on imaging studies for other indications. Symptomatic patients complained of chest pain, cough and dyspnea. All reported cases were located in the anterior or anterior–superior mediastinum and a number of reports document involvement of or an origin from large veins (superior vena cava and innominate vein in particular). The tumours were often partially encapsulated but often at least focally contained infiltrative areas. Mediastinal EHE showed the typical histology with epithelioid endothelial cells, often with intra-cytoplasmic lumina (‘blister cells’), set in a variable hyalinized myxo-collagenous or chondroid matrix. Osteoclastic giant cells and metaplastic bone were reported in several cases [191]. Intra-nuclear cytoplasmic inclusions are another feature typically observed in EHE. A recurrent genetic aberration in EHE, including mediastinal cases, is translocation of the *CAMTA1* gene on chromosome 1p and fusion with the *WWTR1* gene on chromosome 3q [7, 53]. A subset of EHE harbour a different genetic abnormality in which a *YAP1-TFE3* fusion gene is generated from *YAP1* sequences on chromosome 11 and *TFE1* sequences from the X-chromosome [9]. To date, the *YAP1-TFE3* fusion gene has not been reported in mediastinal EHE.

**Fig. 2:**
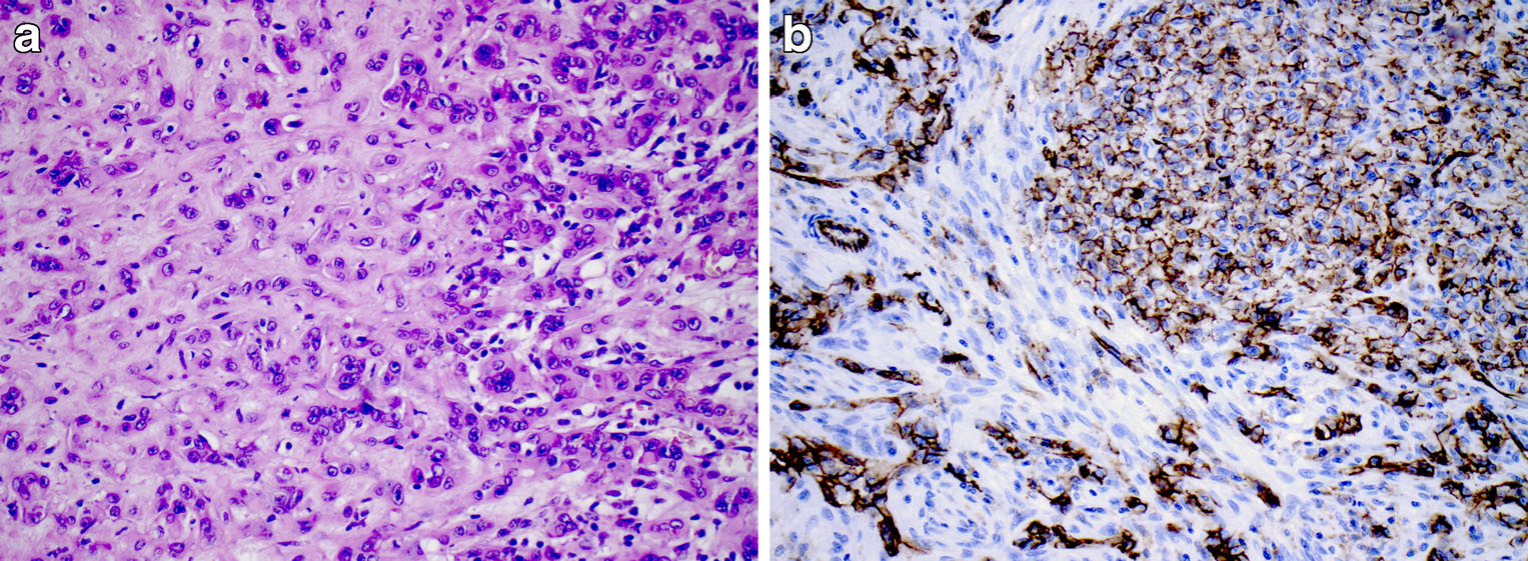
Epithelioid hemangioendothelioma. Anterior mediastinal mass in 56-year-old female. Angiosarcoma was diagnosed on needle biopsy. The tumour was subsequently excised and diagnosed as epithelioid hemangioendothelioma. The presence of a CAMTA1-WWTR1 translocation was confirmed by RT-PCR. The patient remains free of recurrence 23 years post-surgery. **a** The HE stain shows myxo-collagenous matrix with epithelioid cells with intra-cytoplasmic vascular spaces consistent with so-called blister cells. **b** Membranous CD31 staining confirming the vascular nature of the tumour cells

The differential diagnosis of EHE includes epithelioid angiosarcoma (EAS) and metastatic adenocarcinoma. The intra-cytoplasmic lumina in EHE may easily be mistaken for glandular differentiation, which in conjunction with the cytokeratin expression seen in up to 40 % of EHE may lead to an erroneous diagnosis of metastatic carcinoma. The diagnosis of EHE can be established by recognition of the vascular differentiation by immunohistochemistry (with CD31, CD34, ERG or FLI staining) (Fig. 2).

Epithelioid angiosarcoma shows high-grade cytology and usually lacks the typical myxoid, hyaline or chondroid stroma of EHE. Features more commonly associated with EAS include capillary vessels, vascular lakes and papillary growth [7]. The genetic changes identified in the EHE have only been identified in a single case of EAS and thus may serve to distinguish the two entities. The prognosis of mediastinal EHE is not unfavourable; of the 15 cases with follow-up, 13 patients showed no evidence of disease following surgical removal, one patient died of complications of the surgical procedure, and one patient died of the tumour 9 years after initial presentation (no surgery was performed). A subdivision into low- and high-grade EHE on the basis of cytonuclear features has been suggested [7].

**Fig. 3:**
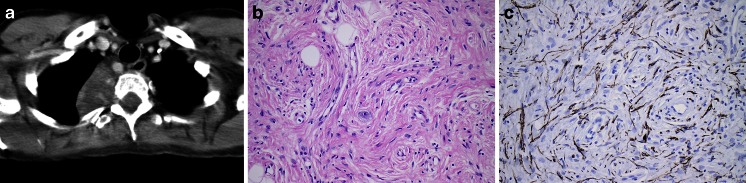
Ganglioneuroma. Paraspinal tumour in the mediastinum of a 45-year-old female, extending from the cervical region to the diaphragm. The mass was excised, the patient remained disease free. **a** CT image showing the cervical aspect of the paraspinal mass. **b** HE stain of the tumour, revealing a ‘schwannian’ background with scattered ganglion cells. **c** Neurofilament stain revealing elongated slender cytoplasmic extensions

Mediastinal AS has almost exclusively been reported in the anterior mediastinum. Less than 40 cases are on record [7, 42, 67, 69, 93, 151, 195, 217, 230]. Although older reports frequently described a favourable outcome even after marginal resection, it should be noted that many of these did not include immunohistochemical confirmation, raising the possibility that the reports may have incorporated entities that would currently not be diagnosed as AS. Furthermore, the prognosis and the demographics of the patients in older series are in marked contrast with more recently described cases. In a recent series of mediastinal EAS, 12 of 13 patients succumbed to the disease [7], whilst in older reports with follow-up data, no evidence of disease was documented in seven of ten patients after surgical removal (follow-up 6–36 months) [42, 67, 69, 93, 195, 217]. Similarly, in a more recent series, there was a distinct male predominance with a median age of 67.5 years, whilst in the older reports, an equal gender distribution with a median age of 42 years was recorded. The demographic and prognostic data of the recent series are more concordant with the high tumour-related mortality reported for AS in other organs [57]. In contrast to EHE, all cases of AS were symptomatic with pain being the most common presenting symptom. Most AS measured less than 10 cm in largest dimension and showed an infiltrative growth pattern. There are too few well-documented cases to draw firm conclusions on treatment and prognosis. The main differential diagnoses are other vascular neoplasms, in particular EHE. However, AS is an infiltrative neoplasm with high-grade cytology and usually lacks the peculiar fibro-myxoid stroma of EHE. It is not uncommon to observe AS as a component of somatic type malignancy in mediastinal germ cell tumours [35, 47, 69, 120, 131, 171, 230].

## Chondro-osseous tumours

To qualify as a primary mediastinal osteo- or chondrosarcoma, an origin from bony structures, chest wall soft tissue, tracheo-bronchial structures and a putative relationship with a germ cell tumour must be excluded. Bearing these restrictions in mind, about ten cases of primary extraskeletal mediastinal osteosarcoma cases occurring in all compartments of the mediastinum have been reported [28, 70, 84, 86, 110, 186, 196, 208, 221]. However, similar to neoplasms described in previous paragraphs, several reports predate current concepts and ideally would require substantiation. Although reported cases spanned a wide age range (19–77 years), six cases occurred in young adults (19–30 years). Four patients were females, and six were males. A single case was most likely a radiation induced osteosarcoma following radiotherapy for Hodgkin’s disease [28]. Most patients presented with chest pain or dyspnea. Synchronous secondary deposits were reported in several cases [110, 186, 196, 221]. Tumours ranged in size from 4.5 to 16 cm and were often partially encapsulated. Of eight patients with follow-up data, five died of disease, one patient was alive with disease and two patients showed no evidence of disease after therapy, including one patient who developed a recurrence, which was treated with radiotherapy. Osteosarcoma has only rarely been reported as somatic type malignancy in germ cell tumours [131, 204].

Less than ten cases of extraskeletal mediastinal chondrosarcoma have been reported, six cases were described in a single series [189] and the remaining cases as case reports. Tumour location in the anterior as well as in the posterior mediastinum was described for both conventional and mesenchymal chondrosarcomas. Patients ranged from 11 to 36 years with no obvious sex predilection [91, 189]. Tumours ranged in size from 5 to 15 cm and were mostly encapsulated. The mesenchymal chondrosarcomas contained areas of primitive small cells and more mature cartilaginous areas, whilst the conventional chondrosarcomas were composed of hyaline cartilage with increased cellularity and atypical chondrocytes. In the cases with follow-up, recurrences occurred in five patients, two patients died of disease (one after a recurrence) and two patients were alive without disease (one after a recurrence). Chondrosarcoma arising in a germ cell tumour appears to be extremely uncommon with only a single reported case in the mediastinum [125].

The histological differential diagnosis of mediastinal osteosarcoma and chondrosarcoma is very limited. Osseous metaplasia may occur in other tumours including thymoma [183] but is unlikely to be confused with the atypical cells of osteosarcoma. Bone or cartilage tissue of variable maturity may be a component of a mediastinal teratoma but does also not show the irregular texture and cellular atypia of osteo- and chondrosarcomas. Osteosarcomatous differentiation has also been reported in malignant peripheral nerve sheath tumours [44].

### Ewing sarcoma/primitive neuroectodermal tumour (PNET)

In the WHO classification, Ewing sarcoma (ES)/PNET is included in the classification of bone tumours despite the fact that up to 20 % of cases are extraskeletal (extra-osseous Ewing sarcoma, eES) and are located in various soft tissue sites and even in parenchymal organs. Furthermore, the cell of origin and differentiation of ES/PNET remain currently unknown. As stated above for chondro-osseous tumours, an origin from bony structures such as vertebrae and chest wall must be excluded in order to qualify as primary mediastinal eES/PNET, leading to the exclusion of several reported cases where tumours extended to these structures or where insufficient details were given [112, 231]. Less than 20 convincing cases of eES/PNET have been reported in the mediastinum [3, 50, 74, 75, 100, 102, 119, 153, 164, 169, 178, 231]. The cases mostly occurred in young individuals (median age 28 years, range 5–66 years) with equal gender distribution and in all compartments of the mediastinum. The presenting symptoms were non-specific; pain and dyspnea were frequent. Some tumours extended through the foramina and caused nerve compression symptoms [100, 119, 169]. The characteristic genetic translocation involving the *EWSR1* gene on chromosome 22 was confirmed in several cases [75, 119]. Multimodal therapy was given in a number of cases with variable results. Long-term cure was achieved in isolated cases.

The differential diagnosis is broad and includes eES/PNET with variant translocations and eES-like tumours unrelated to EWS (small blue round cell tumours), as well as synovial sarcomas and undifferentiated pleomorphic sarcomas with a small cell morphology. In the anterior mediastinum, T lymphoblastic lymphoma (TLBL) is a major differential diagnostic consideration, especially since both tumours express CD99. Immunohistochemical staining for lymphocyte markers and TdT will exclude lymphoma. Small cell neuroendocrine carcinoma (both primary mediastinal and metastatic) can be excluded by its high-grade cytologic features and expression of neuroendocrine and (sometimes weak) epithelial markers and TTF1. In the posterior mediastinum, neuroblastoma must be excluded, which is usually CD99 negative and does not show *EWSR1* gene translocations.

## Nerve sheath tumours and tumours derived from the autonomic nervous system

Neural tumours are the most common mesenchymal tumours in the mediastinum with an estimated incidence of 12–19 % of all mediastinal tumours. Aspects of these tumours have been the subject of several comprehensive reviews [65, 116, 122, 165, 167]; the various entities will therefore only be covered in general terms in this review, for specific details the reader is referred to these reviews. Neural mediastinal tumours may be categorized into tumours of nerve sheath—schwannian derivation, which are predominantly seen in adults and those of the autonomic (neuronal–ganglionic/sympathetic–parasympathetic) nervous system, which are much more common in the paediatric age group. Tumours which are thought to arise form embryonic remnants of the neural tube, such as rare examples of ependymoma (Table 1), constitute a third category [43, 54, 222].

**Table 1:** Summary of mediastinal neural tumours

Neural tumours				
Derivation		Type	Behaviour	Location in mediastinum/age of onset
Nerve sheath/schwannian		Schwannoma	Benign	Post./adult
		Cellular schwannoma	Benign	Post./adult
		Malignant melanotic psammomatous schwannoma	Malignant	Post./adult
		Nerve sheath myxoma	Benign	NA
		Hybrid schwannoma—perineurioma	Benign	NA
		Neurofibroma	Benign	Post./all ages
		Plexiform neurofibroma	Benign	Post./young adult
		MPNST	Malignant	Post./all ages
		Malignant Triton tumour	Malignant	Post./all ages
		Granular cell tumour	Benign	Post./young adult
		Malignant granular cell tumour	Malignant	Post./adult
Ganglionic/autonomous nervous system	Sympathetic ganglia, neuronal/neuroblastic	Ganglioneuromas	Benign	Post./ped./young adult
		Neuroblastoma	Malignant	Post./ped.
		Ganglioneuroblastoma	Malignant	Post./ped.
	Paraganglioma	Anterior mediastinal—branchiomeric–chemodectoma	Benign/malignant	Ant./adult
		Posterior mediastinal paraganglioma (50 % functional/adrenergic)	Benign/malignant	Post./young adult (male)
Embryonal neural tube remnants		Ependymoma	Low-grade malignant	Post./na
Neuroectodermal		Melanotic neuroectodermal tumour	Low-grade malignant	Ped.

Schwannomas are the most common of the mediastinal neural tumours and are considered to arise from spinal nerves. They are therefore mainly seen in the posterior mediastinum, although cases located in the anterior mediastinum are also on record [193, 206]. A significant proportion of cases are asymptomatic and discovered incidentally by routine imaging investigations. Symptomatic cases present with chest pain, cough or compression symptoms, in particular in tumours extending through spinal foramina. There is no gender predilection for schwannoma; they are most common in young- to middle-aged adults. Similar to other locations, these are circumscribed encapsulated tumours, which may undergo cystic degeneration and degenerative (‘ancient’) changes. Surgical removal is curative with extremely low recurrence rates.

The cellular variant of schwannoma, which may be mistaken for a malignant tumour owing to its increased cellularity and lack of Verocay bodies, is particularly common in the mediastinum. Its benign behaviour has been confirmed in several series [58, 113, 218, 223]. A more aggressive variant of schwannoma, previously designated melanotic schwannoma, has been shown to have a high recurrence and metastatic rate with associated 27 % mortality in a single series, prompting the prefix ‘malignant’ to its name [201]. Malignant melanotic schwannoma may be associated with the Carney complex and typically originates from spinal nerves in the posterior mediastinum or retroperitoneum.

A single hybrid nerve sheath tumour sharing features of perineurioma and schwannoma has been reported in the posterior mediastinum. These hybrid tumours are commonly found in superficial soft tissues and require immunohistochemistry to confirm their hybrid nature [157]. Because of their coexpression of vimentin and EMA, they need to be distinguished from meningioma, type A thymoma and follicular dendritic cell sarcoma. A single case of myxoid neurothekeoma/nerve sheath myxoma in the superior mediastinum was recently reported [176].

Neurofibroma, similar to schwannoma, occurs mainly in the posterior mediastinum. Up to 45 % of neurofibromas occur in patients with neurofibromatosis type I (von Recklinghausen’s disease; NF1), in this setting, the tumours occur at a younger age and are often multiple [116]. Plexiform neurofibroma is pathognomonic for NF1 and carries a risk of transformation to MPNST. The anatomical location and ill-defined borders (in contrast to schwannoma) of plexiform neurofibroma hamper removal resulting in a higher recurrence rate.

Malignant peripheral nerve sheath tumours are high-grade sarcomas which typically arise in association with larger nerve trunks and carry a poor prognosis. MPNST, in particular those arising in NF1-associated neurofibromas, can occur in young patients [45, 46]. Similar to other nerve sheath tumours, mediastinal MPNST is typically located in the posterior compartment, although cases may also arise in the anterior mediastinum [92, 99, 148, 166, 232]. Complete surgical resection may provide a cure [105, 124], but overall, these are aggressive neoplasms with high rates of local recurrence and metastases. Variants of MPNST, which have also been reported in the mediastinum, may show divergent mesenchymal differentiation with components of skeletal muscle (rhabdomyosarcoma, the so-called Triton tumour) [105], osteosarcoma and chondrosarcoma [44]. MPNST, including the Triton tumour variant, has been reported as a somatic component of mediastinal germ cell tumours [33, 118, 131].

Granular cell tumour (GrCT) shows differentiation along neuroectodermal lines and shares similarities with Schwann cells. GrCT occurs in the posterior mediastinum, in keeping with its proposed neuroectodermal/neural derivation. Less than 20 cases of GrCT have been reported in the mediastinum [1, 4, 8, 15, 40, 76, 87, 95, 98, 117, 137, 159, 168, 170, 179, 184, 185, 224], including cases with malignant behaviour or atypical features suggestive of malignant GrCT [40, 76, 137, 185]. Benign mediastinal GrCT occurred twice as frequently in females as in males; malignant GrCT (four cases) were equally distributed in males and females. Benign GrCT were seen in young- to middle-aged adults (range 11–53 years, median age 27 years) with an average size of 4 cm and often discovered incidentally. Malignant GrCTs were symptomatic and occurred in older individuals (range 59–66 years, median age 63 years) and were larger (range 5.4–15 cm, average size 10.3 cm).

A very rare tumour with proposed neuroectodermal differentiation based on electron microscopic comparative studies, melanotic neuroectodermal tumour of infancy (retinal anlage tumour, melanotic progronoma), which most commonly occurs in the jaws of infants has twice been reported in the mediastinum [36, 128]. These tumours show a biphasic morphology with small cells of supposed neural origin which are immunoreactive for neuronal/neuroendocrine markers such as synaptophysin and chromogranin, surrounded by larger cells with (neuro)ectodermal differentiation evidenced by staining reactions with EMA, cytokeratin and HMB45. Two cases have been reported in the mediastinum; one case presented in a 7-month-old infant and second case was reported in a 23-year-old female who died of metastases [36, 128].

Neuroblastic tumours with variable ganglionic differentiation originating from sympathetic ganglia range from benign ganglioneuromas through to malignant neuroblastoma and the intermediate form, ganglioneuroblastoma [116]. In line with their postulated cell of origin, most of these tumours develop in the posterior mediastinum. Malignant neuroblastic–ganglionic tumours (neuroblastoma and ganglioneuroblastoma) are very rarely encountered in the adult population [11], whilst ganglioneuroma is seen in children and young adults and is a benign and often asymptomatic tumour (Fig. 3) [116]. Whilst a significant proportion of neuroblastomas present in the mediastinum, it has been suggested that these tumours differ from the more common adrenal neuroblastomas by their pathogenesis and a more favourable prognosis [116]. A single case of neuroblastoma as a somatic component of a mediastinal germ cell tumour was reported, in which the neuroblastemous component metastasized and resulted in the demise of the patient [144].

Mediastinal paraganglionic tumours of the autonomous nervous system have not infrequently been reported in the mediastinum as case series and single case reports, many of which have been included in recent reviews [66, 104, 132, 141, 142, 146, 150]. Mediastinal paragangliomas may occur in the anterior mediastinum, where they are associated with aorticopulmonary, vagal, subclavian, carotid and coronary vessel (branchiomeric) paraganglia. Branchiomeric (aorticopulmonary) paraganglia (chemodectomas) are rarely functional [219].

Paragangliomas located in the posterior compartment derive from the sympathetic chain and are located in the costovertebral sulcus and secrete catecholamines in up to 50 % of cases, resulting in systemic symptoms (‘extra-adrenal pheochromocytoma’). Mediastinal paraganglioma occurs over a wide age range; those located in the anterior mediastinum are slightly more common in females, whilst paravertebral paraganglioma has slightly more frequently been reported in young adult males [66, 132]. The biological behaviour of these tumours cannot reliably be predicted based on morphological features. Up to a quarter of cases may metastasize [104]. Owing to the close vicinity of branchomeric paraganglia to mediastinal vessels and organs and associated surgical considerations, the prognosis of this subgroup is worse than for paravertebral paraganglioma.

Remnants of the embryonic neural tube may on very rare occasions give rise to tumours located in the mediastinum. Six cases of mediastinal ependymoma, including one case of myxopapillary ependymoma, have been published, which were summarized in a case report by Estrozi et al. [54]. All cases occurred in the posterior mediastinum of adult females (age range 35–71 years; median age 40.5 years). The tumours ranged in size from 5.0 to 9.0 cm. Of four cases with follow-up, three patients were alive without disease after surgical resection, and one patient suffered metastases to the pleura and mediastinal lymph nodes.

## Tumours of uncertain differentiation

The WHO classification of soft tissue tumours includes a chapter on neoplasms with unclear histogenesis, some of which have been reported to occur in the mediastinum (Table 2) and are described in the following section. It is conceivable that a number of these tumours may not actually have a normal tissue counterpart but instead reflect the consequence of genetic aberrations in which translocations and other genetic errors culminate in histological patterns, which are not encountered in normal tissue. A number of the entities included in this section of the WHO classification are site-specific, such as intra-muscular myxoma, and are not encountered in the mediastinum; those that have been reported in the mediastinum are listed in Table 2 and are described in the following section. The term ‘malignant mesenchymoma’ which was used in the past for sarcomas with two or more types of differentiation is no longer recommended since the respective tumours are now classified as sarcomas with areas of divergent differentiation (e.g. dedifferentiated liposarcoma or Triton tumour).

**Table 2:** Mediastinal mesenchymal tumours of uncertain differentiation

Tumour type	*N*
Angiomatoid fibrous histiocytoma [12, 38]	2
Ossifying fibromyxoid tumour [49, 197]	2
Myoepithelioma/myoepithelial carcinoma/mixed tumour/parachordoma [10]	1^a^
Synovial sarcoma	<50
Epithelioid sarcoma [71]	1
Alveolar soft part sarcoma [59]	2
Clear cell sarcoma [194, 199]	2
Extraskeletal myxoid chondrosarcoma [189]	1
Extrarenal rhabdoid tumour [73, 155, 158, 198]	5
PEComa [6, 18, 26, 63, 78, 96, 97, 134, 200, 212–214]	<15

^a^Not accepted as true mediastinal origin

### Angiomatoid fibrous histiocytoma

Despite its name, angiomatoid fibrous histiocytoma is generally considered a tumour of uncertain histogenesis rather than a true fibrohistiocytic tumour (see part I of this review). Two cases of angiomatoid fibrous histiocytoma, which were both resected without further disease activity, have been reported in the mediastinum [12, 38].

### Ossifying fibromyxoid tumour

Two cases of ossifying fibromyxoid tumour (OFT) have been reported in the mediastinum, both in the anterior compartment [49, 197]. One case presented as a large mass (1,052 g) in a 50-year-old female who complained of chest pain [197]. No evidence of recurrence or metastasis was noted 1-year after surgery on follow-up. The second case presented as an 11-cm tumour in a 59-year-old female; no follow-up data was provided [49].

### Myoepithelial tumours including parachordoma

There have been no convincing reports of myoepithelial tumours in the mediastinum. A single case of a myoepthelial tumour located in the inferior mediastinum in close association with the carina was suggested to originate from heart muscle and thus cannot be considered a bona fide tumour of mediastinal soft tissue [10].

### Synovial sarcoma

Synovial sarcoma (SySa) is a high-grade spindle cell sarcoma of uncertain histogenesis, often with epithelial differentiation, most commonly occurring in deep soft tissue of the extremities in young adults. SySa is one of the more frequently encountered sarcomas in the mediastinum.

Salah et al. performed a literature survey of mediastinal SySa and identified 40 cases in the English language with appropriate descriptive details [173], including 15 patients described in a series by Suster et al. [190]. The majority of cases occurred in young adults (age range 3–83 years; median age 30.5 years) with a male predominance (2.9:1). Seventy percent of cases were reported in the anterior mediastinum with tumour size ranging from 5 to 20 cm (median size 11 cm). Pain and dyspnea were the most common presenting symptoms.

Most cases were monophasic spindle cell SySa on histology. A specific chromosomal translocation, t(X;18)(p11;q11), involving the *SS18* (*SYT*, *SSXT*) gene on chromosome 18 and the *SSX1*, *SSX2* or *SSX4* gene on the X-chromosome is a characteristic feature of SySa and has been demonstrated in mediastinal SySa [94, 172, 203].

Complete surgical resection was achieved in 57.5 % of patients, which was combined with either radiation or chemotherapy in eight patients. Progression occurred in 67 % of patients. Eleven of 30 (37 %) patients with follow-up data died of disease, nine (30 %) were alive with disease and nine (27 %) patients were alive with no evidence of disease; disease status was unknown for two patients. Complete resection was identified as the sole factor influencing survival.

The differential diagnosis of mediastinal SySa comprises other spindle cell sarcomas, in particular MPNST, malignant solitary fibrous tumour (SFT) and pleomorphic sarcoma NOS. Type A thymoma and sarcomatoid carcinoma may also enter the differential diagnosis.

Type A thymoma is usually composed of uniform non-hyperchromatic epithelial spindle cells, whilst the cells in synovial sarcoma are hyperchromatic and mitotically active. The glandular and epithelial components in biphasic synovial sarcoma may be mistaken for elements of type A thymoma.

Immunohistochemistry is very useful. Although synovial sarcoma is usually positive for cytokeratin and EMA, staining is typically weak and/or focal, whilst type A thymoma is typically diffusely cytokeratin positive. TLE1 and CD56 are commonly expressed in SySa and not found in type A thymoma, whilst bcl2 expression is found in both. Demonstration of the t(X;18) translocation by FISH is diagnostic of SySa, and this genetic abnormality has not been identified in other tumours.

MPNST may be very difficult to distinguish from synovial sarcoma without molecular analysis, as these tumours share some morphological and immunohistochemical features. S-100 staining may be seen synovial sarcoma, and conversely MPNST is frequently completely negative for S-100. Similarly, staining for TLE1 may also be positive in MPNST and SFT. SFT may be distinguished from SySa by CD34 and STAT-6 immunohistochemistry, as both markers are not expressed in SySa.

### Epithelioid sarcoma

A single case of mediastinal epithelioid sarcoma was identified in the literature. The tumour was located in the posterior mediastinum of a 1-year-old male infant. After radiotherapy and chemotherapy, no evidence of disease was recorded after a follow-up period of 44 months [71].

### Alveolar soft part sarcoma (ASPS)

Two well-documented cases of primary mediastinal ASPS have been reported to date. ASPS is a rare tumour with a distinctive histology and characteristic genetic translocation which arises in the deep soft tissue of the lower limb girdle, most commonly in young individuals. ASPS ultimately carries a poor prognosis, and metastases may occur after long time intervals. The two mediastinal cases described in a report by Flieder et al. were located in the anterior mediastinum of a 23-year-old male and in the posterior mediastinum of a 22-year-old male [59]. One of these patients presented with synchronous metastatic disease in the lungs and brain, and the second patient was lost to follow-up after 14 years without evidence of recurrence or metastasis. A third putative case of primary mediastinal ASPS was reported in the anterior mediastinum of a 13-year-old female. However, during work-up, a tumour was later identified in the thigh, which most likely represents the primary location [177].

### Clear cell sarcoma of soft tissue

Two cases of clear cell sarcoma (CCS; melanoma of soft parts) have been reported as primary tumours in the mediastinum [194, 199]. A 15-cm tumour was excised from the superior mediastinum of a 59-year-old female in the report by Tirabosco et al. [199]. Although the histology was representative for CCS, immunohistochemical staining for HMB45 and Melan-A was negative. The diagnosis was supported by demonstration of a translocation involving the *EWSR1* gene by FISH analysis; the partner gene was not identified. Metastatic disease developed 16 months post-surgery; no further follow-up was provided. A second case of CCS was resected by VATS from the superior mediastinum of a 63-year-old male [194]. A tumour of 8.5 cm was removed and showed the typical histology for CCS with positive staining for HMB45 and S-100 protein. Melan-A was negative and no genetic analysis was performed. At 15 months of follow-up, there was no evidence of disease.

### Extraskeletal myxoid chondrosarcoma

One case of mediastinal extraskeletal myxoid chondrosarcoma was included in the series of chondrosarcoma of Suster et al. [189].

### Extrarenal rhabdoid tumour

Extrarenal rhaboid (ERRT) tumour is an exceedingly rare tumour, which is histologically similar to its renal and central nervous system counterparts. ERRT occurs typically in young infants and carries an exceptionally poor prognosis. The *SMARCB1 (INI1)* gene, located on chromosome 22q11, is mutated or deleted in ERRT. Despite its rarity in somatic soft tissue, several cases, mainly included in series, have been reported in the mediastinum [73, 155, 158, 198]. All five patients with sufficient clinical details died of disease. One female patient was diagnosed at the age of 26 years, and two paediatric patients were infants (8 months and a congenital case).

### PEComa

Tumours with assumed perivascular differentiation and an epithelioid morphology (PEComa) include angiomyolipoma, which contains a variable component of mature fat and most commonly arises in the kidney. PEComa cells have a distinct morphology and immunophenotype with evidence of smooth muscle and melanocytic differentiation with variable staining for smooth muscle actin, desmin and melanocytic markers such as Melan-A and HMB45. Since their first description, PEComas have been described in many anatomical locations. PEComa may arise in the setting of tuberous sclerosis and lymphangioleiomyomatosis.

To date, less than 15 cases of PEComa have been reported in the mediastinum, which have all behaved in a benign fashion [6, 18, 26, 63, 78, 96, 97, 134, 200, 212–214]. Three quarters of the reported cases were discovered incidentally by imaging studies for other reasons, symptomatic cases mainly presented with shortness of breath. The marked female predominance seen in non-mediastinal cases was less pronounced in the mediastinal tumours (2:1). The cases were reported over a wide age range (22─63 years; median age 54.4 years). Anatomically, the cases were evenly distributed in the anterior (*n* = 5) and posterior (*n* = 4) mediastinum with single cases in other compartments, with sizes ranging from 4 to 15 cm (average size 7.8 cm). Three of reported cases are likely to have arisen in association with tuberous sclerosis/lymphangioleiomyomatosis [134, 200, 212].

### Desmoplastic small round cell tumour

A single case of desmoplastic small round cell tumour (DSRCT) was reported in the anterior mediastinum of a 22-year-old male [138].

### Unclassified/undifferentiated sarcoma

High-grade undifferentiated sarcomas were previously often classified as malignant fibrous histiocytoma (MFH). Following the current understanding that there is insufficient support for histiocytic (or any other specific) differentiation, these tumours are now categorized as undifferentiated sarcomas in the WHO classification. So-called ‘MFH’ cases have been described in the mediastinum but do not show any further characteristic features. A series of 34 so-called MFH cases in the mediastinum were reviewed by Murakawa et al. [135], and further individual case reports haven been published. As in other soft tissue sites, these tumours are seen over a wide age range but mainly in older adults, with an equal gender distribution. As would be expected, these tumours have a very poor prognosis despite therapeutic intervention.
